# Transposable elements and their potential role in complex lung disorder

**DOI:** 10.1186/1465-9921-14-99

**Published:** 2013-10-05

**Authors:** Muralidharan Sargurupremraj, Matthias Wjst

**Affiliations:** 1 Molecular genetics of lung diseases group, Comprehensive Pneumology Center (CPC), Institute of Lung Biology and Disease (ILBD), Helmholtz Zentrum München, GmbH, Ingolstadter, Landstrasse 1, D-85764, Neuherberg, Munich, Germany

**Keywords:** COPD, Chronic obstructive pulmonary disease, Transposons, Hypomethylation, Genome instability, NGS, Next generation sequencing

## Abstract

Transposable elements (TEs) are a class of mobile genetic elements (MGEs) that were long regarded as junk DNA, which make up approximately 45% of the genome. Although most of these elements are rendered inactive by mutations and other gene silencing mechanisms, TEs such as long interspersed nuclear elements (LINEs) are still active and translocate within the genome. During transposition, they may create lesions in the genome, thereby acting as epigenetic modifiers. Approximately 65 disease-causing LINE insertion events have been reported thus far; however, any possible role of TEs in complex disorders is not well established. Chronic obstructive pulmonary disease (COPD) is one such complex disease that is primarily caused by cigarette smoking. Although the exact molecular mechanism underlying COPD remains unclear, oxidative stress is thought to be the main factor in the pathogenesis of COPD. In this review, we explore the potential role of oxidative stress in epigenetic activation of TEs such as LINEs and the subsequent cascade of molecular damage. Recent advancements in sequencing and computation have eased the identification of mobile elements. Therefore, a comparative study on the activity of these elements and markers for genome instability would give more insight on the relationship between MGEs and complex disorder such as COPD.

## A) Transposable elements and their mobility

Transposable elements (TEs) account for nearly half (approximately 45%) of the human genome, which is in contrast to the functional genes that constitute a smaller proportion (approximately 5%) of the human genome [1]. Based on the mechanism of transposition, TEs are classified as class 2 elements or DNA transposons (‘cut and paste’ mechanism of DNA intermediates) and class 1 elements or retrotransposons (‘copy and paste’ mechanism of RNA intermediates) [1,2]. Of these, retrotransposons are the most important TEs because they can amplify and increase the host genome size. This ability to move enables class 1 elements to strongly affect genome evolution. Retrotransposons are further subdivided into long terminal repeat (LTR) elements and non-LTR elements.

**Figure 1:**
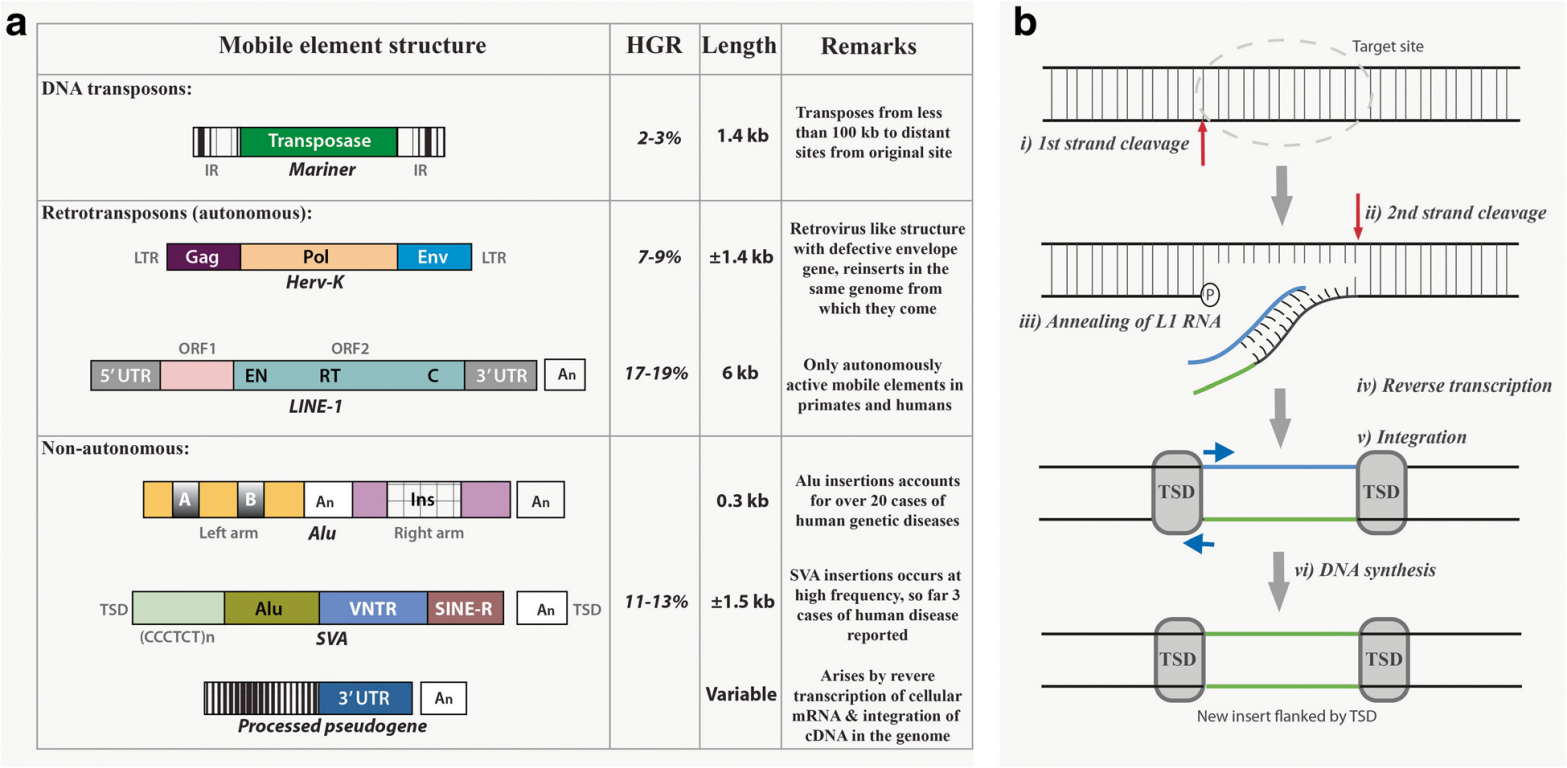
**a) List of mobile elements, structure and its distribution in the human genome.** Reference sequence (HGR) and their structure along with examples (*Italicized*) Abbreviations: IR (Inverted repeats); LTR (Long Terminal Repeat); Gag (Group-specific antigen); Pol (Polymerase); Env (Envelope protein); UTR (Untranslated region); EN (Endonuclease); RT (Reverse transcriptase); C (Cysteine – rich domain); ORF (Open reading frame); A_n_ (Poly (A) tail); A & B (Sequences of RNA pol III promoter); Ins (Insertional sequence); TSD (Target site duplication); VNTR (Variable number of tandem repeats); SINE-R (domain derived from previous translocation). Figure 1**b)** Mobile element insertion by Target Primed Reverse Transcription (TPRT) method. **i**) Endonuclease (EN) coded by transposons cleaves the first DNA strand of the target site; **ii**) Cleavage of the second DNA strand; **iii**) L1 RNA anneals to the nick site; **iv**) Reverse transcription is initiated by retrotransposons coded reverse transcriptase (RT); **v**) Integration; **vi**) DNA synthesis resulting in the new insert with target site duplications at the flanks of newly integrated region.

**Figure 2:**
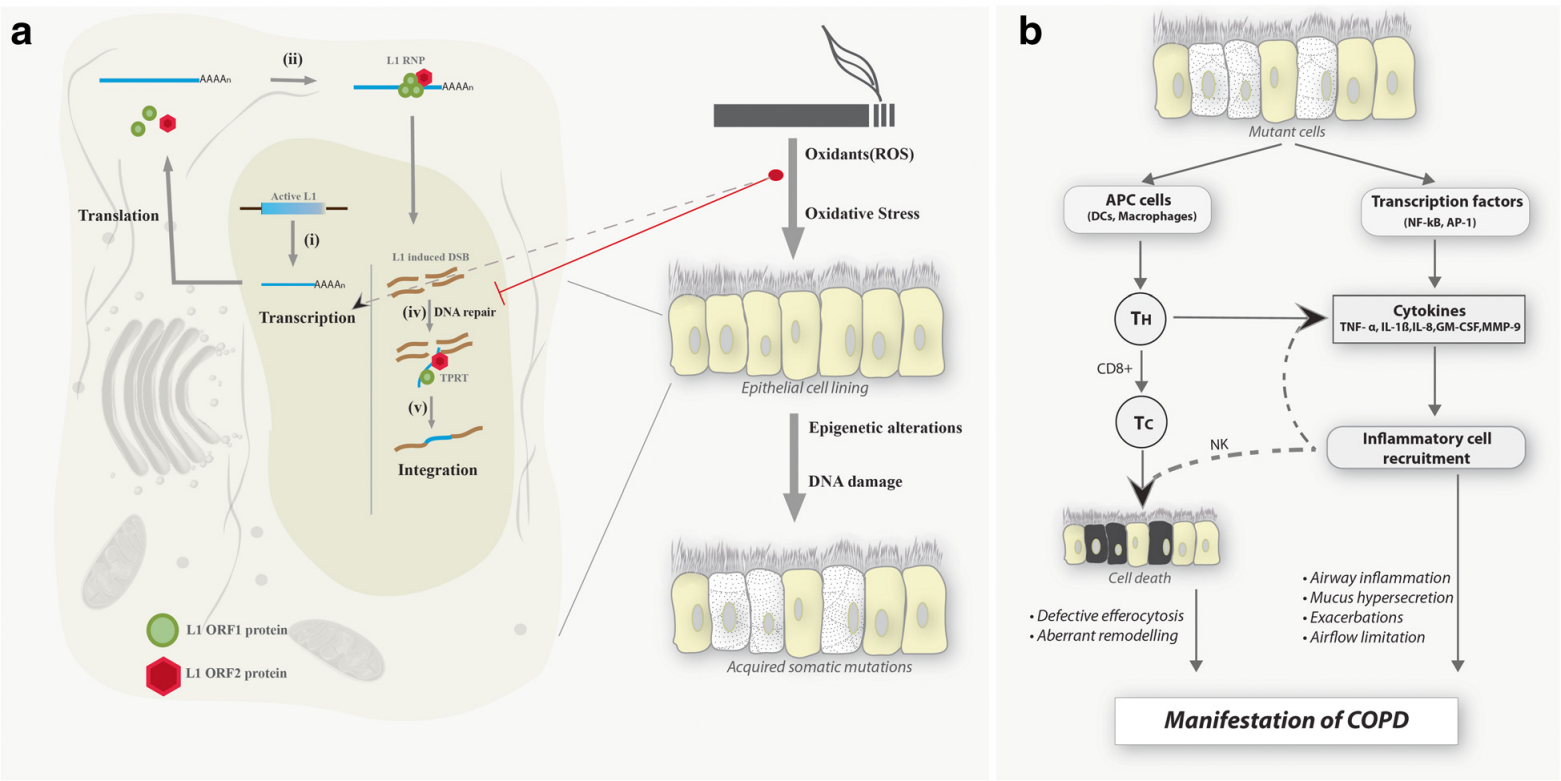
**a. Life cycle of L1 retrotransposon. i**) **Transcription**; L1 life cycle starts with the transcription of active L1 in the genome by recruitment of transcription factors, followed by polyadenylation and splicing to form L1 RNA, which is nuclear exported. **ii**) **Translation**; Active L1RNA codes for the ORF1 and ORF2 protein that binds with other retrotranscription competent L1 (RC-L1) RNA to form L1RNP (Ribonucleoprotein) complex, which is nuclear imported for retrotransposition. **iii**) **Insertional events**; results in DSBs by the activity of L1 ORF2 endonuclease followed by, **iv**) **integration**; lesions created by L1ORF2 activity is repaired and integrated in to the genome by TPRT. v) Heavy metals and other smoke particles can interact with L1 lifecycle either at the early stages by altering the methylation profile (epigenetic alteration) resulting in active L1 or at the late repair stages by impairing repair pathway resulting in somatic mutation accumulation (*Granulated cells*). **b.** Effect of somatic mutation accumulation on disease onset and exacerbation. Mutated somatic cells are recognized by the host system as foreign cells and are presented by antigen presenting cells (APCs) triggering a cascade of pathways involving T helper cells (Th) and cytotoxic T cells (Tc), which migrates to the infected site and releases various transmitters inducing cell death. Failure in effective efferocytosis results in aberrant remodeling of the structure and the characteristic onset of COPD. Mutant cells can interact with transcription factors to increase the release of cytokines and the consequent recruitment of inflammatory cells thereby destabilizing the immune balance and manifest the features of COPD.

Long interspersed nuclear elements (LINEs) are non-LTR elements that lack LTRs at their ends. Most LINEs belong to the LINE-1 (or L1) family and are the only TEs capable of transposing autonomously, which constitute approximately 17% of the human genome [1,3]. Although majority of L1s are rendered inactive as molecular fossils by 5′ truncations and inversions [4], there are still approximately 80–100 active retrotransposition-competent L1s (RC-L1s). An active L1 is approximately 6 kb in length, containing a 5′-UTR, 2 open reading frames (ORF1 and ORF2) and a 3′-UTR with the characteristic poly (A) tail (Figure 1a) [3,5]. L1 elements either have *cis* or *trans* preference [6]. Proteins coded by L1s with *cis* preference (ORF1p and ORF2p) act on other L1 RNAs to aid nuclear import and integration into the genome (Figures 1b, 2a) [7]. Proteins coded by L1s with *trans* preference assists in the translocation of other non-autonomous elements such as short interspersed nuclear elements (SINEs) (Figure 1a) [6].

## B) Transposition and genome instability

Genome integrity is a crucial determinant in passing down genetic information from one generation to another. TE-associated genetic alterations such as aberrant mRNA splicing, introduction of premature stop codon and transcriptional disruptions threaten this integrity. Double-stranded breaks (DSBs) generated by TEs [8] produce tracts of non-allelic sequences that can derange major homology-based repair system (homologous recombination repair, HRR). This in turn can result in large-scale insertion/deletions (INDELS), inversions and chromosomal rearrangements through non-allelic homologous recombination (NAHR) [9]. Thus far, more than 25 insertion-mediated disorders have been reported [10]. Furthermore, TEs play a crucial role in the genesis of structural variations such as microsatellites repeats [11]. For instance, before integration, L1s and SINEs undergo 3′ extension to generate a 3′-A-rich tail [3,5], which directs further integration of TEs [12]. These newly integrated retrotransposons can readily mutate to pro-microsatellite sequences and turn in to highly unstable structures by processes such as polymerase slippage [13], resulting in microsatellite instability (MSI). Such an association has been reported in microsatellite-initiating mobile elements (mini-me) of dipteran taxa [14] that carry pro-microsatellite sequences. After the insertion of mini-me into the genome, slippage-associated mutation introduces variation in these loci to generate microsatellites. The mechanism observed in dipteran genomes seems to be common among eukaryotes where elements with cryptic repeats tend to decay into microsatellites through insertion-mediated mutations [14].

Microsatellites exhibit high mutation rate compared to point mutations, which makes them a potent regulator of gene expression [15]. MSI, a type of genomic instability, is a modulator in several malignant and benign diseases caused by the instability in tandem repeats (2–6 bp) of microsatellites [16]. MSI is studied by amplifying microsatellites that are proximal to a putative gene and examining the shift in electrophoretic pattern caused by the addition or deletion of repetitive units [17]. Genetic studies on MSI have already shown its implications as acquired mutations in benign lung conditions [18] and as a potential marker for asthma, chronic obstructive pulmonary disease (COPD) and idiopathic pulmonary fibrosis [17,19,20]. Epithelial cells lining the trachea, bronchi and bronchioles of the lungs are prone to such mutations [21]. These mutations can persist even after smoking cessation, possibly explaining the non-intractable inflammation condition in ex-smokers. Studies on bronchial epithelium of smokers [22] further validate this theory of epithelium cells as the prime cells of MSI activity. Furthermore, MSI is significantly associated with exacerbation frequency in patients with COPD [23]. COPD exacerbation is caused by the acute worsening of respiratory symptoms along with physiological deteriorations. Because its frequency is related to disease severity [24], the possible role of MSI in regulating this frequency should be an interesting avenue to study.

## C) Transposable elements and complex lung disorder

COPD is a complex lung disorder and is the leading cause of morbidity and mortality. The 2011 WHO estimates indicate that 64 million people have COPD; moreover, COPD is reported to cause 3 million deaths worldwide, making it the fifth leading cause of death worldwide [25]. COPD manifests as co-occurrence of conditions such as chronic bronchitis (inflammation of the bronchi) and emphysema (alveolar wall destruction) [26]. Cigarette smoking is the most common cause of COPD and is associated with inflammation, high cell turnover and oxidative stress, leading to proteolytic damage of the lungs. Nearly all smokers develop inflammation, but only a fraction (10%–15%) develop COPD and even fewer (1%–3%) develop lung cancer [21]. This peculiar distribution urges one to postulate that acquired (somatic) mutations may be a prerequisite in the pathobiology of COPD. Estimates show that genetic alterations accounts for up to 50% of COPD cases [27]. Marked variability in the development of airflow obstruction among smokers [28], familial aggregation of pulmonary function in monozygotic and dizygotic twins [29], and differences in clinical outcome compared with controls in first-degree relatives [30] are some of the facts that support the claim of genetic factors in COPD development. In addition, linkage and candidate gene association studies have identified an array of genetic determinants in the pathogenesis of COPD [26]. Although there are reports on genomic instability events in complex disorders such as COPD and cancer [15,31], the association of these events with TE activity remains obscure. Therefore, it is possible that TEs such as L1s may play a vital role in disease phenotype by introducing somatic mutations and thereby affecting genome integrity.

TEs can be acquired as somatic mutations over a lifetime; presence of L1 activity in tumour cells but not in the surrounding healthy cells supports this hypothesis [32]. Propagation of TEs in the somatic line is facilitated by their expansion in germ cells or in the embryonic stage. In addition, retrotransposition events occurring in germ cells greatly increase the chance of TE propagation to further generations [33]. For instance, family studies on ocular disease show that mothers of patients exhibit both somatic and germline mosaicism for L1 insertion in the disease gene, suggesting the possibility of retrotransposition during embryogenesis [34]. Retrotransposition events occurring during developmental stages can create somatic mosaicism. Kano et al. (2009) studied such occurrences where L1 RNA was found in embryonic cells and adult tissues such as the lung [35]. Further quantitative analysis showed that frequency of retrotransposition was higher in somatic tissues as in reproductive cells. A recent study supports this claim because in this study, the level of L1 RNA in the oesophagus and lung was same as that in HeLa cells [36]. Ever increasing results from molecular studies on transgenic models emphasise the risk of such genetic alterations in the development of organs. It is possible that active L1-mediated retrotransposition can disrupt the genes that regulate lung growth in early life, resulting in developmental deformity. This may further lead to lung damage by host machinery (protease/anti-protease imbalance) or by environmental factors (cigarette smoking, pollutants). For instance, it is already known that epigenetic changes during lung development play a vital role in the development of bronchopulmonary dysplasia (BPD) [37] and that any associated lower lung functions can ultimately result in the development of COPD [38].

## D) Epigenetics of transposable elements

The study of heritable non-coding variations is a hot topic, particularly in cancer biology. DNA methylation is one such epigenetic regulator that plays a decisive role in developmental biology and pathobiology by processes such as X-chromosome inactivation and retrotranscription silencing [39]. Approximately one-third of the DNA methylation occurs in mobile elements such as Alu and L1s [40], thus making them inactive and surrogate markers of global methylation analysis. These sites can be hypomethylated by environmental influences, leading to genome instability and altered gene expression [41]. Reports on the association between global hypomethylation and genomic instability [20] suggest that L1s that are hypomethylated in airway epithelial cells are associated with higher levels of microsatellite instability. A recent study supports this hypothesis by showing the association between hypomethylation of L1 elements and faster rate of decline in lung function measures such as FEV1 and FVC [42]. Because lung function tests are a major determining factor for diagnosing lung disorders and measuring their severity, the impact of hypomethylation on lung function is intriguing. Other environmental factors such as wood smoke exposure may also contribute in this type of association [43]. Environmental factors are a known source of oxidative stress, and any associated epigenetic alterations at the microsatellite level manifests as acquired mutations, resulting in MSI incidence [44]. Such instability events have already been studied in COPD patients by examining the by-product of oxidant-DNA damage [8-hydroxydeoxyguanosine (8-OHdG) marker] [31].

## E) Oxidative stress and hypomethylation

In recent years, there has been an interest in studying the effects of oxidative stress on epigenetic gene regulation by DNA methylation. Oxidative stress caused by oxidant/anti-oxidant imbalance plays a central role in the pathogenesis of COPD [45]. Oxidant release results in the inactivation of anti-proteases, neutrophil sequestration and gene expression of pro-inflammatory cytokines. Cigarette smoke is an exogenous source of such oxidants that contain a high proportion of free radicals, both in tar and gaseous phase. The smoke interacts with the epithelial lining fluid to form cigarette smoke condensate, which in turn produces more reactive oxygen species [46]. In addition, under stress, inflammatory cells (neutrophils and macrophages) can act as endogenous source of oxidants, which in turn damage the components of lung matrix (emphysema) by proteolytic cleavage [45].

Under oxidative conditions, GC-rich sites are highly susceptible, and guanine with the lowest redox potential [47] oxidizes to guanyl neutral radical. These neutral radicals react with superoxides from cigarette smoke to form 8-OHdG [48]. 8-OHdG, a stable oxidation product, inhibits the binding capacity of DNA methyltransferase, resulting in the demethylation of guanine [49] and cytosine residues [50]. Furthermore, 8-OHdG can cause transversions (G > T) that reduce methylation hotspots (CpG dinucleotides), leading to more hypomethylation [51]. Because the susceptibility to oxidative stress depends on the base composition, clusters of GC-rich CpG dinucleotides can serve as major targets. For instance, the L1 mRNA is bicistronic (ORF1 and ORF2) in nature, with 5′-UTR having a high GC content (approximately 60%) [52,53]. In one study on bladder cancer, patients with increased oxidative stress exhibited hypomethylation of L1 elements [54]. Similarly, global methylation analysis on lung adenocarcinoma samples showed hypomethylation of L1s that resulted in increased mobility and subsequent gene disruption [20]. Oxidative stress-induced demethylation can be a result of environmental factors such as smoke exposure, ageing, UV radiation and lifestyle factors. For instance, prenatal exposure to tobacco smoke is significantly associated with global (L1s and Alu) demethylation in adulthood [55]. In addition, cigarette smoking along with the inhalation of traffic particles decreases the methylation of L1 in blood DNA [56]. All these studies point to oxidative stress and its role in the methylation pattern of TEs. Under oxidative stress, these sites can undergo hypomethylation, resulting in the activation and transposition of L1s (Figure 2a); this can lead to deleterious structural alterations in the genome (mutant cells) [41] followed by a cascade of signalling events (Figure 2b). Such events can bring in cell death and/or inflammatory response with a continuous cycle of inflammation leading to continued decline of lung function. All these studies clearly suggest that these are not isolated events in the development of COPD and that oxidative stress mediated epigenetic changes plays a central role in the pathogenesis.

## F) Identification of transposable element activity in the genome

Marked variability in the distribution of active TEs between individuals is a direct consequence of their activity in somatic tissues and low selection pressure encountered by these elements. It enables them to evolve rapidly at different sites that make their identification in the genome arduous. Over the last 2 decades, new approaches have been applied for identifying mobile elements. Earlier studies mostly used previous knowledge of mutant genes in characterizing the mobile elements by cloning and sequencing [11,57], which was further refined by the advent of tools such as PCR [58]. The sheer complexity and vast distribution of these elements makes their identification a mammoth task, with massive data pouring in from new applications such as next-generation sequencing (NGS).

**Figure 3:**
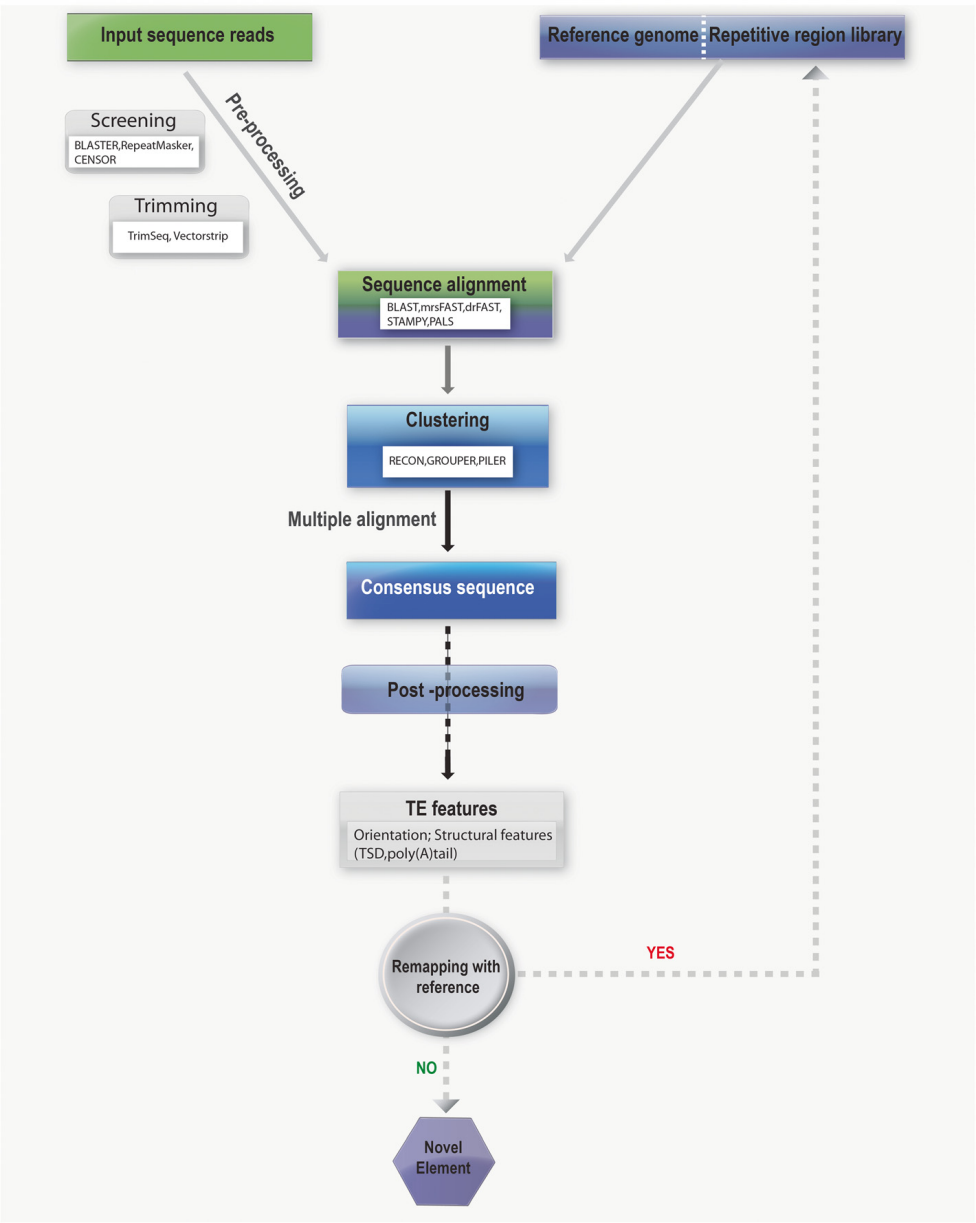
**General scheme of pipeline in identifying repeated sequences.** (*White inset boxes* – *Few examples of the available computational tools*) Input query sequence data, is pre-processed by screening for TEs and the cryptic structures (poly (A) tail, degenerate primers) are trimmed to avoid excessive mismatches. It is then mapped against the reference genome and/or repeat region library to form clusters, for each cluster the programs (MAP, MAFFT) constructs multiple alignments resulting in consensus sequences. Followed by a post processing step wherein the consensus is realigned with the reference by using characteristic TE features as filter parameters, yielding concordant (YES) or discordant combinations (NO). Concordant combinations are the elements that are already in the reference library while the discordant combinations are of much interest as it represents putative novel elements.

A few of these methods such as *de novo* discovery and homology-based methods are briefly discussed. The algorithm for detecting inserts in *de novo* method usually involves reading shotgun sequence reads and matching the repeat sequences, followed by clustering the matched pairs to give a consensus sequence of a TE family [59]. Unlike the *de novo* sequencing method, homology-based approach uses previous knowledge of TE sequences, such as sequence similarity, in identifying similar class TEs with a low copy number. Figure 3 discusses the main theme of computational study in repetitive elements; putative L1 insertions are identified by comparing clusters of consensus alignment from the same sequence reads. A sequence pair read that is aligned to the reference genome is concordant; hence, discordant alignment that does not match paired-end expectations could represent novel structural variant (SV) sites [60]. Recent studies enhanced the sensitivity and specificity of this procedure by using refined versions of the algorithm that targets the diploid nature of the genome [61]. As a valuable addition to the sequence paired-end read alignment, Ewing et al. (2010) used the orientation and structural characteristics of the reads to identify 1016 novel L1 insertions [62].

Research interest in SVs has increased exponentially over the past decade, and with the advent of screening technologies, approximately 5000 insertions have been reported thus far [63]. Because most reported insertions are scattered across other databases leading to redundancy, a compiled non-redundant list is eminent. Database of Retrotransposition Insertion Polymorphism (DbRIP) represents a comprehensive list of human genome variations (SINE, Alu and LINE). Data from published journals are collected and compiled into a non-redundant list of RIPs. The design of the database is based on simple genome browser style with graphical visualization of RIP for easy navigation and information retrieval. Classification of reported RIPs is based on class, family and subfamily, including data on the size of insertion, chromosomal position, disease association and PCR conditions with expected amplicon sizes and reference(s). Such a tool, with effective documentation, gives a much clearer picture of RIPs in the line of SNPs and CNVs. Now, with the advent of next-generation platform and organized data, it is possible to study the role of these elements in shaping the genome structure and their functional impact.

## G) Summary and concluding remarks

At least 4 principal mechanisms, inflammation, protease-anti-protease imbalance, oxidative stress and apoptosis, have been identified in the pathogenesis of COPD. Of these, the oxidative stress plays a pivotal role in COPD pathogenesis because it directly injures the respiratory tract and regulates other mechanisms.

Oxidative stress elicits inflammatory response and inhibits the DNA repair system in a dose-dependent manner that may be altered at the microsatellite level, resulting in genome instability. The vast distribution and complexity of mobile genetic elements in the genome makes another strong argument in genomic instability. In addition to acting as insertional mutagens, alterations such as deletions, inversion and duplication can be attributed to the translocation of these active mobile elements. Studies on lung barrier epithelial cells have proven the effect of airway inflammation and oxidative stress on genome instability. Upon exposure to cigarette smoke, barrier epithelial cells undergo epigenetic alterations that can trigger mobile elements such as L1s, thereby influencing multiple molecular pathways that enhance inflammatory signals. Novel L1 sites can be identified by performing whole genome analysis of epithelial cell DNA from smokers (COPD), ex-smokers (no COPD) and healthy controls against a reference genome. Such new L1 insertions can be compared against the profiles of microsatellite markers in patient samples to study the relationship between mobile genetic elements and genome instability and their potential role in a complex disorder such as COPD.

## Abbreviations

TEs: Transposable elements; MGEs: Mobile genetic elements; LINEs: Long interspersed nuclear elements; DSBs: Double strand breaks; HRR: Homologous recombination repair; NAHR: Nonallelic homologous recombination; MSI: Microsatellite instability; BPD: Bronchopulmonary dysplasia; COPD: Chronic obstructive pulmonary disease; FEV1: Forced expiratory volume in 1 second; FVC: Forced vital capacity; RIP: Retrotransposon insertion polymorphism; CNVs: Copy number variants.

## Competing interests

The authors declare that they have no competing interests.

## Authors’ contributions

MW is responsible for suggestions and final revision of the draft. MS is responsible for the concept and design of the paper, preparation of the draft and take full responsibility for the final version of this manuscript. Both authors read and approved the final manuscript.

## References

[B1] Lander ES Linton LM Birren B Nusbaum C Zody MC Baldwin J Devon K Dewar K Doyle M FitzHugh W Funke R Gage D Harris K Heaford A Howland J Kann L Lehoczky J LeVine R McEwan P McKernan K Meldrim J Mesirov JP Miranda C Morris W Naylor J Raymond C Rosetti M Santos R Sheridan A Sougnez C Initial sequencing and analysis of the human genome Nature 2001 409 860 921 10.1038/35057062 11237011

[B2] Belancio VP Roy-Engel AM Deininger PL All y'all need to know 'bout retroelements in cancer Semin Cancer Biol 2010 20 200 210 10.1016/j.semcancer.2010.06.001 20600922 PMC2943028

[B3] Beck CR Garcia-Perez JL Badge RM Moran JV LINE-1 elements in structural variation and disease Annual review of genomics and human genetics 2011 12 187 215 10.1146/annurev-genom-082509-141802 21801021 PMC4124830

[B4] Ostertag EM Kazazian HH Twin priming: a proposed mechanism for the creation of inversions in L1 retrotransposition Genome Res 2001 11 2059 2065 10.1101/gr.205701 11731496 PMC311219

[B5] Ostertag EM Kazazian HH Jr Biology of mammalian L1 retrotransposons Annual review of genetics 2001 35 501 538 10.1146/annurev.genet.35.102401.091032 11700292

[B6] Wei W Gilbert N Ooi SL Lawler JF Ostertag EM Kazazian HH Boeke JD Moran JV Human L1 retrotransposition: cis preference versus trans complementation Mol Cell Biol 2001 21 1429 1439 10.1128/MCB.21.4.1429-1439.2001 11158327 PMC99594

[B7] Cost GJ Feng Q Jacquier A Boeke JD Human L1 element target-primed reverse transcription in vitro EMBO J 2002 21 5899 5910 10.1093/emboj/cdf592 12411507 PMC131089

[B8] Gasior SL Wakeman TP Xu B Deininger PL The human LINE-1 retrotransposon creates DNA double-strand breaks Journal of molecular biology 2006 357 1383 1393 10.1016/j.jmb.2006.01.089 16490214 PMC4136747

[B9] Gilbert N Lutz-Prigge S Moran JV Genomic deletions created upon LINE-1 retrotransposition Cell 2002 110 315 325 10.1016/S0092-8674(02)00828-0 12176319

[B10] Callinan PA Batzer MA Retrotransposable elements and human disease Genome and Disease 2006 1 104 115 10.1159/000092503 18724056

[B11] Arcot SS Fontius JJ Deininger PL Batzer MA Identification and analysis of a ‘young‘ polymorphic Alu element Biochimica et Biophysica Acta (BBA)-Gene Structure and Expression 1995 1263 99 102 10.1016/0167-4781(95)00080-Z 7632743

[B12] Nadir E Margalit H Gallily T Ben-Sasson SA Microsatellite spreading in the human genome: evolutionary mechanisms and structural implications Proc Natl Acad Sci USA 1996 93 6470 6475 10.1073/pnas.93.13.6470 8692839 PMC39047

[B13] Hebert ML Wells RD Roles of Double-strand Breaks, Nicks, and Gaps in Stimulating Deletions of CTG.CAG Repeats by Intramolecular DNA Repair Journal of molecular biology 2005 353 961 979 10.1016/j.jmb.2005.09.023 16213518

[B14] Wilder J Hollocher H Mobile elements and the genesis of microsatellites in dipterans Mol Biol Evol 2001 18 384 392 10.1093/oxfordjournals.molbev.a003814 11230539

[B15] Wooster R Cleton-Jansen AM Collins N Mangion J Cornelis RS Cooper CS Gusterson BA Ponder BA von Deimling A Wiestler OD Instability of short tandem repeats (microsatellites) in human cancers Nat Genet 1994 6 152 156 10.1038/ng0294-152 8162069

[B16] Charlesworth B Sniegowski P Stephan W The evolutionary dynamics of repetitive DNA in eukaryotes Nature 1994 371 215 220 10.1038/371215a0 8078581

[B17] Siafakas NM Tzortzaki EG Sourvinos G Bouros D Tzanakis N Kafatos A Spandidos D Microsatellite DNA instability in COPD CHEST Journal 1999 116 47 51 10.1378/chest.116.1.47 10424502

[B18] Samara KD Tzortzaki EG Neofytou E Karatzanis AD Lambiri I Tzanakis N Siafakas NM Somatic DNA alterations in lung epithelial barrier cells in COPD patients Pulmonary pharmacology & therapeutics 2010 23 208 214 10.1016/j.pupt.2009.12.001 20040382

[B19] Zervou MI Tzortzaki EG Makris D Gaga M Zervas E Economidou E Tsoumakidou M Tzanakis N Milic-Emili J Siafakas NM Differences in microsatellite DNA level between asthma and chronic obstructive pulmonary disease Eur Respir J 2006 28 472 478 10.1183/09031936.06.00127305 16707512

[B20] Rabinovich EI Kapetanaki MG Steinfeld I Gibson KF Pandit KV Yu G Yakhini Z Kaminski N Global methylation patterns in idiopathic pulmonary fibrosis PLoS One 2012 7 e33770 10.1371/journal.pone.0033770 22506007 PMC3323629

[B21] Anderson GP Bozinovski S Acquired somatic mutations in the molecular pathogenesis of COPD Trends in pharmacological sciences 2003 24 71 76 10.1016/S0165-6147(02)00052-4 12559770

[B22] Wistuba II Mao L Gazdar AF Smoking molecular damage in bronchial epithelium Oncogene 2002 21 7298 7306 10.1038/sj.onc.1205806 12379874

[B23] Makris D Tzanakis N Damianaki A Ntaoukakis E Neofytou E Zervou M Siafakas NM Tzortzaki EG Microsatellite DNA instability and COPD exacerbations Eur Respir J 2008 32 612 618 10.1183/09031936.00169307 18508815

[B24] Makris D Moschandreas J Damianaki A Ntaoukakis E Siafakas NM Milic EJ Tzanakis N Exacerbations and lung function decline in COPD: new insights in current and ex-smokers Respiratory medicine 2007 101 1305 1312 10.1016/j.rmed.2006.10.012 17112715

[B25] World Health Organization Factsheet No.315 2012 [http://www.who.int/mediacentre/factsheets/fs315/en/index.html]

[B26] Silverman EK Progress in chronic obstructive pulmonary disease genetics Proc Am Thorac Soc 2006 3 405 408 10.1513/pats.200603-092AW 16799082 PMC2658703

[B27] Coultas DB Hanis CL Howard CA Skipper BJ Samet JM Heritability of ventilatory function in smoking and nonsmoking New Mexico Hispanics Am J Respir Crit Care Med 1991 144 770 775 10.1164/ajrccm/144.4.770 1928947

[B28] Burrows B Knudson RJ Cline MG Lebowitz MD Quantitative relationships between cigarette smoking and ventilatory function The American review of respiratory disease 1977 115 195 205 842934 10.1164/arrd.1977.115.2.195

[B29] Redline S Tishler PV Rosner B Lewitter FI Vandenburgh M Weiss ST Speizer FE Genotypic and phenotypic similarities in pulmonary function among family members of adult monozygotic and dizygotic twins Am J Epidemiol 1989 129 827 836 2923128 10.1093/oxfordjournals.aje.a115197

[B30] Khoury MJ Beaty TH Tockman MS Self SG Cohen BH Familial aggregation in chronic obstructive pulmonary disease: use of the loglinear model to analyze intermediate environmental and genetic risk factors Genet Epidemiol 1985 2 155 166 10.1002/gepi.1370020206 3876967

[B31] Tzortzaki EG Dimakou K Neofytou E Tsikritsaki K Samara K Avgousti M Amargianitakis V Gousiou A Menikou S Siafakas NM Oxidative DNA damage and somatic mutations: a link to the molecular pathogenesis of chronic inflammatory airway diseases Chest 2012 141 1243 1250 10.1378/chest.11-1653 22116800

[B32] Miki Y Nishisho I Horii A Miyoshi Y Utsunomiya J Kinzler KW Vogelstein B Nakamura Y Disruption of the APC gene by a retrotransposal insertion of L1 sequence in a colon cancer Cancer Res 1992 52 643 645 1310068

[B33] Ostertag EM DeBerardinis RJ Goodier JL Zhang Y Yang N Gerton GL Kazazian HH A mouse model of human L1 retrotransposition Nature genetics 2002 32 655 660 10.1038/ng1022 12415270

[B34] van den Hurk JA Meij IC Seleme MC Kano H Nikopoulos K Hoefsloot LH Sistermans EA de Wijs IJ Mukhopadhyay A Plomp AS de Jong PT Kazazian HH Cremers FP L1 retrotransposition can occur early in human embryonic development Hum Mol Genet 2007 16 1587 1592 10.1093/hmg/ddm108 17483097

[B35] Kano H Godoy I Courtney C Vetter MR Gerton GL Ostertag EM Kazazian HHJ L1 retrotransposition occurs mainly in embryogenesis and creates somatic mosaicism Genes Dev 2009 23 1303 1312 10.1101/gad.1803909 19487571 PMC2701581

[B36] Belancio VP Roy-Engel AM Pochampally RR Deininger P Somatic expression of LINE-1 elements in human tissues Nucleic Acids Res 2010 38 3909 3922 10.1093/nar/gkq132 20215437 PMC2896524

[B37] Merritt TA Gadzinowski J Mazela J Adamczak AM Epigenetic influences in the development of bronchopulmonary dysplasia Archives of Perinatal Medicine 2011 17 17 22

[B38] Roos AB Berg T Nord M A Relationship between Epithelial Maturation, Bronchopulmonary Dysplasia, and Chronic Obstructive Pulmonary Disease Pulmonary Med 2012 2012 1 10 10.1155/2012/196194 PMC3540891 23320163

[B39] Issa J-P Opinion: CpG island methylator phenotype in cancer Nat Rev Cancer 2004 4 988 993 10.1038/nrc1507 15573120

[B40] Kochanek S Renz D Doerfler W DNA methylation in the Alu sequences of diploid and haploid primary human cells The EMBO journal 1993 12 1141 1151 8384552 10.1002/j.1460-2075.1993.tb05755.x PMC413315

[B41] Wilson AS Power BE Molloy PL DNA hypomethylation and human diseases Biochim Biophys Acta 2007 1775 138 162 17045745 10.1016/j.bbcan.2006.08.007

[B42] Lange NE Sordillo J Tarantini L Bollati V Sparrow D Vokonas P Zanobetti A Schwartz J Baccarelli A Litonjua AA Alu and LINE-1 methylation and lung function in the normative ageing study BMJ Open 2012 2 e001231 10.1136/bmjopen-2012-001231 23075571 PMC3488751

[B43] Sood A Petersen H Blanchette CM Meek P Picchi MA Belinsky SA Tesfaigzi Y Wood smoke exposure and gene promoter methylation are associated with increased risk for COPD in smokers American journal of respiratory and critical care medicine 2010 182 1098 1104 10.1164/rccm.201002-0222OC 20595226 PMC3001253

[B44] Tzortzaki EG Siafakas NM A hypothesis for the initiation of COPD Eur Respir J 2009 34 310 315 10.1183/09031936.00067008 19648516

[B45] Cantin A Crystal RG Oxidants, antioxidants and the pathogenesis of emphysema Eur J Respir Dis Suppl 1985 139 7 17 2995106

[B46] Chow CK Cigarette smoking and oxidative damage in the lung Ann NY Acad Sci 1993 686 289 298 10.1111/j.1749-6632.1993.tb39189.x 8512254

[B47] Devasagayam TP Steenken S Obendorf MS Schulz WA Sies H Formation of 8-hydroxy(deoxy)guanosine and generation of strand breaks at guanine residues in DNA by singlet oxygen Biochemistry 1991 30 6283 6289 10.1021/bi00239a029 2059635

[B48] Misiaszek R Crean C Joffe A Geacintov NE Shafirovich V Oxidative DNA damage associated with combination of guanine and superoxide radicals and repair mechanisms via radical trapping J Biol Chem 2004 279 32106 32115 10.1074/jbc.M313904200 15152004

[B49] Franco R Schoneveld O Georgakilas AG Panayiotidis MI Oxidative stress, DNA methylation and carcinogenesis Cancer Lett 2008 266 6 11 10.1016/j.canlet.2008.02.026 18372104

[B50] Turk PW Laayoun A Smith SS Weitzman SA DNA adduct 8-hydroxyl-2'-deoxyguanosine (8-hydroxyguanine) affects function of human DNA methyltransferase Carcinogenesis 1995 16 1253 1255 10.1093/carcin/16.5.1253 7767994

[B51] Kuchino Y Mori F Kasai H Inoue H Iwai S Miura K Ohtsuka E Nishimura S Misreading of DNA templates containing 8-hydroxydeoxyguanosine at the modified base and at adjacent residues Nature 1987 327 77 79 10.1038/327077a0 3574469

[B52] Dmitriev SE Andreev DE Terenin IM Olovnikov IA Prassolov VS Merrick WC Shatsky IN Efficient translation initiation directed by the 900-nucleotide-long and GC-rich 5' untranslated region of the human retrotransposon LINE-1 mRNA is strictly cap dependent rather than internal ribosome entry site mediated Mol Cell Biol 2007 27 4685 4697 10.1128/MCB.02138-06 17470553 PMC1951496

[B53] Rogozin IB Kochetov AV Kondrashov FA Koonin EV Milanesi L Presence of ATG triplets in 5' untranslated regions of eukaryotic cDNAs correlates with a 'weak' context of the start codon Bioinformatics 2001 17 890 900 10.1093/bioinformatics/17.10.890 11673233

[B54] Patchsung M Boonla C Amnattrakul P Dissayabutra T Mutirangura A Tosukhowong P Long interspersed nuclear element-1 hypomethylation and oxidative stress: correlation and bladder cancer diagnostic potential PLoS One 2012 7 e37009 10.1371/journal.pone.0037009 22615872 PMC3352860

[B55] Flom JD Ferris JS Liao Y Tehranifar P Richards CB Cho YH Gonzalez K Santella RM Terry MB Prenatal smoke exposure and genomic DNA methylation in a multiethnic birth cohort Cancer Epidemiol Biomarkers Prev 2011 20 2518 2523 10.1158/1055-9965.EPI-11-0553 21994404 PMC3559183

[B56] Baccarelli A Wright RO Bollati V Tarantini L Litonjua AA Suh HH Zanobetti A Sparrow D Vokonas PS Schwartz J Rapid DNA methylation changes after exposure to traffic particles Am J Respir Crit Care Med 2009 179 572 578 10.1164/rccm.200807-1097OC 19136372 PMC2720123

[B57] Kazazian HH Wong C Youssoufian H Scott AF Phillips DG Antonarakis SE Haemophilia A resulting from de novo insertion of L1 sequences represents a novel mechanism for mutation in man Nature 1988 332 164 166 10.1038/332164a0 2831458

[B58] Miki Y Katagiri T Kasumi F Yoshimoto T Nakamura Y Mutation analysis in the BRCA2 gene in primary breast cancers Nat Genet 1996 13 245 247 10.1038/ng0696-245 8640237

[B59] Li R Ye J Li S Wang J Han Y Ye C Yang H Yu J Wong GK ReAS: recovery of ancestral sequences for transposable elements from the unassembled reads of a whole genome shotgun PLoS Comput Biol 2005 1 e43 10.1371/journal.pcbi.0010043 16184192 PMC1232128

[B60] Hormozdiari F Alkan C Eichler EE Sahinalp SC Combinatorial algorithms for structural variation detection in high-throughput sequenced genomes Genome Res 2009 19 1270 1278 10.1101/gr.088633.108 19447966 PMC2704429

[B61] Hormozdiari F Hajirasouliha I Dao P Hach F Yorukoglu D Alkan C Eichler EE Sahinalp SC Next-generation VariationHunter: combinatorial algorithms for transposon insertion discovery Bioinformatics 2010 26 i350 i357 10.1093/bioinformatics/btq216 20529927 PMC2881400

[B62] Ewing AD Kazazian HH High-throughput sequencing reveals extensive variation in human-specific L1 content in individual human genomes Genome Res 2010 20 1262 1270 10.1101/gr.106419.110 20488934 PMC2928504

[B63] Liang P Tang W Database documentation of retrotransposon insertion polymorphisms Front Biosci (Elite Ed) 2012 4 1542 1555 22201974 10.2741/479

